# One Secret of Longevity: The Fatty Acid Pathway

**DOI:** 10.1371/journal.pbio.1001030

**Published:** 2011-03-15

**Authors:** Richard Robinson

**Affiliations:** Freelance Science Writer, Sherborn, Massachusetts, United States of America

**Figure:**
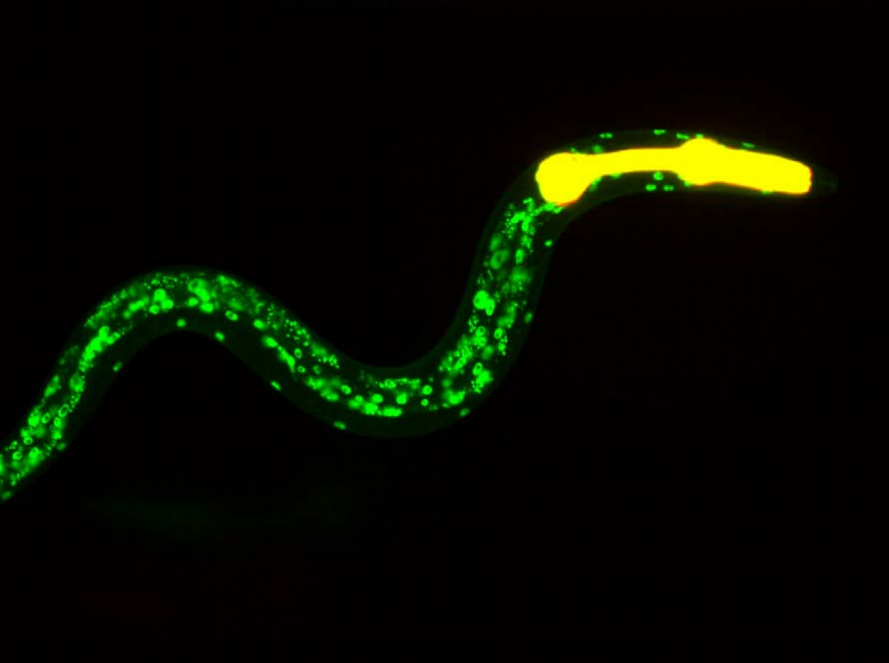
Overexpression of the longevity-promoting gene, *nhr-80* (green), further extends the lifespan of germline-less *C. elegans*. Image credit: Jérôme Goudeau.

Dorian Gray had his picture, Ponce de Leon his fountain. For roundworms and fruit flies, the key to longer life is removing their germ cells—the stem cells that make eggs and sperm. For reasons still unknown, a roundworm without its germ cells lives 60% longer than one with those cells intact. And you don’t even need to actually remove them—mutations that impair their proliferation also extend life. In this issue of *PLoS Biology*, Jerome Goudeau, Hugo Aguilaniu, and colleagues explore the molecular pathways regulating life extension, and show a key role is played by a transcription factor that, among other things, promotes desaturation of fatty acids.

The authors began their exploration by searching for nuclear receptors that were required for the life-extension effect, reasoning that one or more such receptors were likely to play a key role in controlling longevity pathways. Using RNAi in long-lived worms with a known germline proliferation mutation, they shut down hundreds of receptors, looking for those that would reverse the lifespan extension.

They found only one, called *nhr-80*, which they showed acted specifically within the germline-mediated longevity pathway, and not any of the several other pathways known to extend lifespan. This result provided the first experimental evidence that lifespan extension through ablation of the germline can occur in the absence of insulin signaling, one of the best characterized pathways affecting lifespan. Mutation of *nhr-80* to prevent expression of the gene prevented the longevity effect, while overexpression increased it, in both cases only when germline cells were absent.

They next examined candidates for interaction with the *NHR-80* protein (denoted NHR-80), and showed that the nuclear hormone receptor DAF-12, but not the corresponding cytochrome P450, DAF-9, was required for NHR-80 to prolong lifespan. The exact nature of their interaction—whether they bind directly, or through intermediaries—remains unclear.

As a transcription factor, NHR-80 regulates gene expression. Its known targets include a group of enzymes involved in lipid metabolism called desaturases. Desaturases convert saturated fatty acids, such as stearic acid, into unsaturated fatty acids, such as oleic acid, a conversion that significantly alters the physical and biological properties of the fats made from them. The authors show that genes for several desaturases are highly expressed in long-lived germline mutants. As a result, the amount of oleic acid is increased by germline ablation. When the *nhr-80* gene was silenced, the increased expression of one desaturase gene in particular, called *fat-6*, was abolished.

Important biological pathways often have redundancy built into them, so it was not too surprising when the authors found that deletion of *fat-6* alone did not reverse the lifespan extension in germline mutants. However, simultaneously deleting *fat-6* and another desaturase, *fat-7*, did decrease mutant worm longevity, an effect that could be mitigated by adding the enzymes’ end product, oleic acid.

While worms are not humans, we share many of the same cellular mechanisms, and it is possible the same life-extending effects of this germline-loss/fatty acid increase pathway will be found in us. If so, it may be possible to imagine, in some far-off future, that the human analog of *nhr-80* will be the molecular key to the fountain of youth. But if the analogy holds, the next step will be to understand how to promote longevity without giving up one’s fertility!

**Goudeau J, Bellemin S, Toselli-Mollereau E, Shamalnasab M, Chen Y, et al. (2011) Fatty Acid Desaturation Links Germ Cell Loss to Longevity Through NHR-80/HNF4 in** ***C. elegans*** **. doi:10.1371/journal.pbio.1000599**

